# Why Forced-Choice and Likert Items Provide the Same Information on Personality, Including Social Desirability

**DOI:** 10.1177/00131644231178721

**Published:** 2023-06-12

**Authors:** Martin Bäckström, Fredrik Björklund

**Affiliations:** 1 Lund University, Sweden

**Keywords:** forced-choice, ipsative data, social desirability, psychometrics, personality measurement

## Abstract

The forced-choice response format is often considered superior to the standard Likert-type format for controlling social desirability in personality inventories. We performed simulations and found that the trait information based on the two formats converges when the number of items is high and forced-choice items are mixed with regard to positively and negatively keyed items. Given that forced-choice items extract the same personality information as Likert-type items do, including socially desirable responding, other means are needed to counteract social desirability. We propose using evaluatively neutralized items in personality measurement, as they can counteract social desirability regardless of response format.

Brown and Maydeu-Olivares (2011) showed that it is possible to recover latent traits from forced-choice questionnaires. The following research will attempt to show why this is possible and how this can be accomplished with a much simpler methodology. In theory, it should not be possible to know anything about the absolute level of a trait based on the comparison between traits. When someone prefers one trait or behavior over another, we only get information about the relative position of the traits. However, as Brown and Maydeu-Olivares (2011) showed in their seminal paper, even when information about the absolute level is missing the correlation structure in a typical normative rating scale can be estimated by means of structural equation modeling (SEM). Here we will show that it is not necessary to use SEM or Thurstonian Item Response Theory models to recover latent traits. The information is part of the response format used. In other words, it is not a question of recovering; it is rather just a question of extracting the information.

The forced-choice format is often presented as a superior alternative to the ordinary single-stimulus (Likert-type scale) format used in, for example, NEO-PI-R (Costa & McCrae, 1992). Brown and Maydeu-Olivares (2011) argue that many of the biases that plague single-stimulus format Likert-type inventories can be avoided by using multidimensional forced-choice (MFC) format inventories. This claim seems doubtful. It is true that social desirability increases correlation between scales in typical single-stimulus inventories (e.g., Bäckström et al. 2009; Bäckström & Björklund, 2020). But importantly, if they can handle social desirability, why does the SEM model so closely reproduce the correlations between personality variables when MFC items are used? Given that they reproduce the same structure, then there is a high risk that MFC inventories also include the biases they are claimed to counteract.

A major merit attributed to forced-choice inventories is that they handle social desirability, understood as the interaction with the rater’s style of responding. Social desirability can be interpreted either as a content factor that represents a common trait, for example, the general factor of personality (GFP), or a specific trait of personality (impression management, self-enhancing, and/or an evaluative factor). In the first case, the GFP is a higher order factor that influences all personality factors (of the Big-Five). In the second case, social desirability is a factor that biases personality measures but is still referring to personality, it is a sixth trait (Bäckström & Björklund, 2014). Yet another possible interpretation is that social desirability is a measurement factor, a style of responding that is not related to individual differences outside of the measurement situation. It affects and biases the measures, but may be controlled for using other means, for example, measures of the social desirability method factor or by changing the items such that they don’t tap into this rating style.

There has been extensive interest recently in the forced choice method. Among the issues discussed are the relative benefits of the multi-unidimensional pairwise preference model (Stark et al., 2005) and the generalized graded unfolding model (Roberts et al., 2000). The present study will not delve into the debate concerning how to create and estimate forced-choice inventories. Instead, like Brown (2016), Bürkner (2022), and Schulte et al. (2021), we focus on conceptual issues. More precisely, we investigate whether MFC items and Likert-type items have the same information, and whether it is possible to represent the information, for example, correlation structure, from Likert-type inventories using forced choice item sets. Previous studies have compared the forced-choice and single-item format and concluded that the differences are not very large (e.g., Bürkner, 2022; Lee et al., 2018; Schulte et al., 2021; Watrin et al., 2019). Often, they have been based on very complex models (e.g., Zhang et al., 2020), but even if they have succeeded in finding similar reliability and validity, they have not explained why they are so similar. The question we would like to answer is whether there has been an unnecessary separation of MFC methods and single item methods, whether they really measure the same thing and whether there is a simple explanation of why. This issue has also been addressed by Schulte et al. (2021) who showed that the similarity is high and that one explanation could be that the MFC format has more information about the personality traits than expected. The next section will include a detailed evaluation of the MFC format.

## The MFC Format

There are different formats for MFC scales, but the present work will primarily concern the format based on three-item comparisons of different scales. In this response format raters are asked to select one item as most typical of themselves and one as least typical. In this way, the three items are ranked from best, over in between, to worst. Brown and Maydeu-Olivares show that this can be coded by three dummy variables (see Table 1).

**Table 1.** Binary Coding of a Multidimensional Forced-Choice Item With Three Ordered Items Using Three Dummy Variables.

Choices	Dummy1	Dummy2	Dummy3
Item 1 best, Item 3 worst	1	1	1
Item 1 best, Item 2 worst	1	1	0
Item 2 best, Item 3 worst	0	1	1
Item 2 best, Item 1 worst	0	0	1
Item 3 best, Item 2 worst	1	0	0
Item 3 best, Item 1 worst	0	0	0

In addition, items can be either positively keyed or negatively keyed. To mix items that are keyed positively with items keyed negatively is important in this context because it creates more possible comparisons. For example, if you only have two traits (say extraversion and openness) and only positively keyed items, then for all participants with a preference for extraversion over openness, every item will result in the same result—the score for extraversion will be increased and the score for openness will decrease. This creates a dependence between the two variables. In a situation where the preference for extraversion and openness is the exact same, there’s a tie. Then the selection must be solved by randomly selecting one over the other or by doing a more fine-grained analysis. If there are 10 extraversion/openness comparisons, perhaps five will favor extraversion and five for openness so that we end up with the score of five for each trait. However, if positively and negatively keyed items are mixed, the situation changes. Now ties can occur for more than one reason. When there is a strong preference for one item and very little preference for another item that is reversed, then the preference for the item can be a tie. For example, “I am very friendly” and “I don’t like to read intellectual books” could be a tie. So even when there are very different preferences for two items the data format can lead to a tie, and of course, since the preference is a tie, making a random choice will be part of the response. When you have both positively and negatively keyed items the recovering process will be more effective because there is more information available about absolute differences. Schulte et al. (2021) have suggested that it is not possible to use mixed items as “positive items” (e.g., measuring extraversion) always are more desirable and more often selected than “negative items” (e.g., measuring introversion). If such items are mixed, it would be possible for the raters to describe themselves in a desirable manner. However, this is not necessarily so, as it is possible to create evaluatively neutral items (Bäckström et al., 2009) that are similar in desirability when measuring both extraversion and introversion.

### Illustrative Examples

The following examples will hopefully clarify the difference between positive and mixed item-sets (more formal descriptions can be found in Bürkner, 2022; Schulte et al., 2021) by explicating how we could evaluate and/or assign points on different outcomes of an MFC rating using mixed items. Imagine that Jill is a conscientious person who is also an extrovert on exactly the same level. Now Jill is asked if she wants to help organize her schoolbooks or if she wants to hang out and meet friends. She would not be able to choose but does anyway (because that’s how forced-choice item sets work, we do not give her a point, the next time she is confronted with the same type of comparison she may make the opposite choice). Then, we ask her if she wants to take part in a challenge or if she wants to have a nice evening at home. For this choice, she would accept the challenge (she seems conscientious, but not introverted). Next, we ask her if she would consider skipping homework or if she wants to go to an exciting club. She will naturally choose the latter (she seems to be extroverted but not non-conscientious). For both Extraversion and Conscientiousness, she gets two points, she chose the challenge, and she chose the exciting club, in addition she chose the introverted behavior (−1 to be reversed since we are measuring extraversion) and the non-conscientious behavior (−1 point, which must be reversed).

Now imagine Jack, who is neither conscientious nor extroverted. On the first item, he reluctantly responds that he wants to meet friends (he gets no points for this, the choice was random, and a new similar comparison may lead to the opposite choice). On the second item, he chooses to have a nice evening at home, and on the third, he can consider skipping homework. On the traits conscientiousness and extraversion, he gets −2, he chooses the evening at home, which is negative in relation to extraversion (−1) and he skips the homework, negative for conscientiousness (−1), in addition, he chooses the conscientious behavior (1, but it should be reversed) and the extroverted (1 to be reversed). Here it can be seen that, although Jill and Jack both have the equal level of both extraversion and conscientiousness, the way of asking allows us to measure the levels of their traits on both traits at the same time. The response is graded. For the sake of clarity, they would of course also be asked if they want to have a nice evening at home or if they want to skip homework (but they would not be able to choose, so it doesn’t give points). If they were completely neutral, average in relation to conscientiousness and extraversion, they would get zero points, they would never be able to choose.

Now contrast this scenario with one where only positively keyed items are used. Jill is extroverted but not conscientious, she would then choose to hang out with friends, and she automatically becomes more extroverted than conscientious, but we do not find out anything about the level, just that they are different. If she receives 10 such comparisons, she gets 10 points for extraversion and −10 (or zero of you like) for conscientiousness, the size of the difference doesn’t matter, the two traits are related. Then we think of Jack, he is as extroverted as Jill, but also conscientious, to the same extent. Jack would score an average of 0 on both Extraversion and Conscientiousness. Jill would appear conscientious, but Jack would appear average in both traits. The problem is diminished when many scales are estimated at the same time (Bartram, 1996; Schulte et al., 2021), but this has other obvious drawbacks, for example, you always need to measure many scales.

How points are distributed is depicted in Table 2 for a comparison of two items, as in the illustrative example above (see Schulte et al., 2021, for a similar model). The preference for the items is shown using numbers from 1 to 5. Of course, these preferences are like Likert-type ratings, but this can be defended, because when a respondent compares two items, a rating of 4 for one item suggests that it should be selected with higher preference compared with a rating of 2 or 3 for another item. The table includes reversed items, when the same item is used in a negatively keyed version. In the table the preference has been reversed for these items (e.g., 4 is reversed to 2, and 2 is reversed to 4). When there are both positively and negatively keyed items there are four different possible comparisons, positive against positive (I1-I2), negative against negative (I1M-I2M), positive against negative (I1-I2M), and negative against positive (I1M-I2). In the table, a higher preference gives the variable 1 point and a lower preference −1. When there is a tie, both items are given 0 points. For comparison, the two last columns of Table 2 show how points are distributed when only positively keyed items are used, in that case there is only one possible comparison.

**Table 2.** Comparisons of Two Items Using Mixed and Only Positively Keyed Items.

Type	Both positive and negative				Only positive	
	I1	I2	I1M	I2M	I1	I2
Preference	4	2	2	4	4	2
I1-I2	1	−1			1	−1
I1M-I2M			−1	1		
I1-I2M	0			0		
I2-I1M		0	0			
Sum	2	−2			1	−1
Preference	5	2	1	4	5	2
I1-I2	1	−1			1	−1
I1M-I2M			−1	1		
I1-I2M	1			−1		
I2-I1M		1	−1			
Sum	4	0			1	−1
Preference	5	1	1	5	5	1
I1-I2	1	−1			1	−1
I1M-I2M			−1	1		
I1-I2M	0			0		
I2-I1M		0	0			
Sum	2	−2			1	−1
Preference	3	3	3	3	3	3
I1-I2	0	0			0	0
I1M-I2M			0	0		
I1-I2M	0			0	0	
I2-I1M		0	0			0
Sum	0	0			0	0
Preference	4	4	2	2	3	3
I1-I2	0	0			0	0
I1M-I2M			0	0		
I1-I2M	1			−1	0	
I2-I1M		1	−1			0
Sum	2	2			0	0

*Note.* I1 = Item 1; I2 = Item 2; I1M = Reversed Item 1; I2M = Reversed Item 2; Preference = preference score for the two items; Sum = sum score for Item 1 and Item 2.

The upper pane of Table 2 has ratings of 4 and 2 for Items 1 and 2, respectively. If you add together all possible comparisons between these two items, the result will be 2 for Item 1 and −2 for Item 2 (i.e., 1+−(−1)) and (−1 +−(1)) see upper pane of Table 2). When the preference is 5 and 2, the outcome of the four possible comparisons will be 4 and 0 for Items 1 and 2, respectively, and preferences of 3 and 3 will give an outcome of 0 and 0. However, the outcome of the comparisons is complicated on some levels. For example, when one item is ranked 5 and the other 1, the outcome is again 2 and −2. A very important comparison is when both outcomes are highly ranked. For example, when they are 5 and 5, the outcome will be 2 and 2. On the contrary, when both outcomes are ranked low, the outcome will be −2 and −2. All this suggests that there is more information about levels when mixing positively and negatively keyed items in an MFC. All possible outcomes of two items ranked between 1 and 5 are shown in Table 3.

**Table 3.** All Possible Item Preference Comparisons and Outcomes.

Comparisons		Outcome	
Item 1	Item 2	Item 1	Item 2
1	1	−2	−2
2	2	−2	−2
3	3	0	0
4	4	2	2
5	5	2	2
2	1	0	−4
3	1	0	−4
4	1	0	−4
5	1	2	−2
3	2	0	−4
4	2	2	−2
5	2	4	0
4	3	4	0
5	3	4	0
5	4	4	0

The correlation between the Likert-type style item rating and outcome is .86 for both Items 1 and 2. Schulte et al. (2021) also showed that mixed items include information about levels. They conclude that levels are represented in the coding, but that it is not possible to know which variable is higher when using mixed MFC sets. This is obvious from Table 3, where the second item generally has lower values. A possible solution is to balance the placement of scales in the MFC set such that items from all scales has been in all position the same number of times. To accomplish such a balance with a large number of scales, you need to have many item sets.^1^

Even if you add levels to the response format, for example, increase to 100 levels, the correlation between single-stimulus response and the sum of the binary comparisons is very strong (*r* = .82 for 100 levels). To summarize, there is a lot more information about item levels when mixed items are used as MFC item sets.

What if the single-stimulus inventory only has positively keyed items? To replicate the simulations of the present research is easy. It is possible to create binary MFC variables using mixed items just by reversing the responses of the single-stimulus inventory. However, this does not solve the problem when constructing forced-choice inventories. In that case, you need evaluatively neutral items.

## The Present Study

In the empirical section of this study, single-stimulus Likert-type inventories based on real data will be compared with simulated MFC inventories created based on the same single-stimulus inventories. The simulations will use information from the single-stimulus ratings of two different Big-Five inventories and from these create MFC items based on comparisons from how the three scales were rated. Basically, three items from three different scales will be randomly selected and the ratings from the three items will be used to create the information for the three binary variables. Thereafter, three new items are randomly selected, and three new binary variables will be created, and so on. In this way it is possible to create a very large number of MFC items as it is possible to compare many single-stimulus ratings in different ways.

To investigate the possibility to extract the same information, the single-stimulus rating scales will be compared with the simulated MFC scales. Both MFC scales based on positively keyed items and mixed items, that is, with both positively and negatively keyed items, will be included. The main results will be based on artificially reversed items, that is, all items in the simulations are positively keyed from the start, and in the mixed simulations, items from the whole inventory will be reversed randomly. We also test whether using only the factual item keys affects the results. One simulation compares random MFC items and single items and to what extent they can reliably replicate the correlations between scales of an inventory. Finally, we vary the correlation between items within a scale and the correlation between scales using simulated single-item data.

One of our goals is to show that a simple summing of the binary values (here coded as 1 and −1) can be used to calculate the trait values of the MFC scales. This is equivalent to classical coding of forced choice, where selected items are assigned 1 point and not selected are assigned 0 or −1. In addition, the SEM estimation constructed by Brown and Maydeu-Olivares (2012) will be used to test whether the SEM can reproduce the information in the inventory (this replicates part of Schulte et al., 2021). The difference between the SEM-estimated models and the models presented in this study is that the SEM models weigh the items based on how well they fit in with other items from the same scale. One advantage of our coding is that it is similar to the SEM model, but the model presented here simply weighs all items equally (similar to fixing them to 1). In other words, the SEM models are based on estimations that try to optimally weigh the items while the classical coding model just sums the values from the three binary variables constructed from the comparisons.

One problem with MFC scales is to select the best combination of items to compare. To make the MFC items “fair” in relation to the scales, the combination of items from different scales must be optimized, i.e., the MFC scales should consist of a combination of items from all possible scale combinations. If the number of scales is large the number of combinations needed will be very large. If there are 5 scales then the number of unique combinations is 10, and the number of binary variables is 30. If the number of scales is 20 the number of combinations increases to 380 and then the number of binary variables will be 1,140.

## Method

### Data and Materials

Two Five-factor inventories will be used in the present study. One is the International Personality Item Pool (IPIP)-300 inventory (Goldberg et al., 2006) and was created to mimic the NEO-PI-R (Costa & McCrae, 1992). It consists of 300 items that are rated on a five-step Likert-type response scale (from 0 to 4 in this study). The inventory measures the five factors extraversion, agreeableness, conscientiousness, neuroticism, and openness. It also includes 30 facets, six for each factor. Data from 831 respondents will be used.

The second instrument is the Neutralized Big-Five inventory (NB5I, Bäckström et al., 2023) which is a version of the IPIP-300 with a reduced number of facets (20) and only 120 items. The inventory has evaluatively neutralized items (Bäckström et al., 2009), to minimize the correlation between factors and secondary loadings/relations between facets and factors, such that facets of a factor only correlate with its factor and not with other factors in the inventory. Data from 3,229 respondents will be used. All data and R-scripts can be downloaded at https://osf.io/cgzha/?view_only=6606e43ef3434b489f2e568298bbfe30.

### Procedure

To create the binary variables from the simulated MFC items an R function was designed. The function takes responses from a single-stimulus inventory and creates a combination of items from three scales (the minimum number of scales is three). The selection of items from the inventory scales is at random, with replacement. After selecting three items, for each respondent (row in the data frame), it compares the Likert-type ratings, and when the rating is higher for one item than for the other two items it is selected as the one that would have been preferred. The item with the lowest rating is selected as the one rejected. To handle ties, a small random number with a mean of 0 and an *SD* of 0.1 is added to the ratings. This ensures that items from each scale has the same chance of being selected when ratings are the same, that is, a tie. Of course, as there are only five response options, such ties will be rather common.

In the NB5I the mean ratings in the population are close to the middle of the rating scale, and an advantage in this context is that the comparisons will result in a rather even number of selections. In other words, as the mean of all items is almost the same, the chance is about equal that a rating will be higher (or lower) for one item compared to another. If there are large differences in mean ratings some items are more popular and will therefore be selected more often, while others will almost never be selected. This will make the IPIP-300 less suitable in the present study but still possible to use.

The function can create balanced sets of three items from any number of scales. In other words, the combination of items is selected such that items from all scales are combined optimally with items from all other scales. The placement of the items in the item set is also important, as it influences the mean values when the binary variables are summarized. Therefore, the function also balances where items from different factors are placed in the item set (first, second, or third). In this research, we will only use balanced sets of items. It is possible to vary the number of items, for example, to create five sets of balanced MFC items and thereby create very large numbers of items (e.g., one simulation uses 1,000 item sets).

To test whether it is possible to extract the same information from MFC scales as from single-stimulus scales, a few parameters will be varied. These are the number of MFC items created, the number of scales estimated, and whether the simulated MFC instrument includes only positively keyed items or also has mixed items. In addition, randomly generated MFC items will be compared with randomly selected Likert-type items, with regard to how reliably they can represent the underlying data.

## Results

### A First Simulation

To start off the empirical section, a small example simulation will be presented, together with a more detailed report of the procedure and analyses. In the other sections, we will present summary data from the simulations.

The first simulation was based on data for the five Big-five factors of the NB5I inventory, with 3,229 respondents. The simulation mimics the one in Brown and Maydeu-Olivares (2011). In their example, 20 sets of items from five scales were used and the same will be used here. With the 20 sets, we created 60 binary variables so that every scale was estimated with 12 binary variables. We combined the scales in the 20 sets optimally, so all scales were combined with all other scales.

The correlations between scales of the original data, the random ipsative data using weights of 1 (RIp_1), and the random ipsative data estimated using SEM (RIp_SEM)^2^ are shown in Table 4. It is obvious that the correlations based on RIp_1 and RIp_SEM do not closely follow the original data, but there are some similarities. The original single-stimulus correlations are generally low. The RIp_1 tends to have negative correlations between all scales, while the RIp_SEM has rather large both negative and positive correlations between scales.

**Table 4.** Positively Keyed Items: Correlations Between Original Data (N = 3229), the Random Ipsative Data Weighted 1 and the Random Ipsative Data Estimated Using SEM.

Variable	Extraversion	Agreeableness	Conscientiousness	Neuroticism
Agreeableness	−.01			
Conscientiousness	−.02	.11		
Neuroticism	.10	.08	−.18	
Openness	−.02	.12	.10	.00
Agreeableness_1	−.31			
Conscientiousness_1	−.30	−.18		
Neuroticism_1	−.24	−.22	−.33	
Openness_1	−.30	−.19	−.10	−.29
Agreeableness_SEM	−.31			
Conscientiousness_SEM	.46	−.80		
Neuroticism_SEM	.35	−.33	.41	
Openness_SEM	.48	−.76	.75	.42

*Note.* SEM = structural equation modeling.

Table 5 shows the correlations between the original data and the RIp_1 and RIp_SEM estimations. The two highlighted diagonals show that the ipsative data correlate strongly with the original. The only exception is for RIp_SEM and Agreeableness. It is also obvious that the off-diagonal correlations follow the correlations between variables in the original data much better than correlations within RIp_1 and RIp_SEM.

**Table 5.** Positively Keyed Items: Correlations Between Original Data and Random Ipsative Data.

Variable	Extraversion	Agreeableness	Conscientiousness	Neuroticism	Openness
Extraversion_1	.78	−.24	−.17	−.13	−.15
Agreeableness_1	−.21	.66	−.11	−.16	−.17
Conscientiousness_1	−.28	−.15	.71	−.34	−.12
Neuroticism_1	−.13	−.11	−.31	.81	−.20
Openness_1	−.29	−.09	−.06	−.25	.70
Extraversion_SEM	.56	−.51	.01	−.10	.03
Agreeableness_SEM	.22	.17	−.32	.25	−.23
Conscientiousness_SEM	−.13	−.43	.53	−.22	.13
Neuroticism_SEM	−.06	−.42	−.08	.60	.00
Openness_SEM	−.12	−.40	.18	−.18	.53

*Note.* SEM = structural equation modeling.

Brown and Maydeu-Olivares (2011) showed that using a mix of positively keyed and negatively keyed items resulted in a much better reproduction of the original correlations. In the introduction, it was shown that there is much more information in ipsative data with mixed items than data based on items keyed in only one direction.

Tables 6 and 7 show the results of estimations using mixed items. Table 6 reveals that the low correlations between the variables in the original data is reproduced in both the RIp_1 data and RIp_SEM data. Table 7 shows that the correlations between variables in the original data are very strong in both the RIp_1 and the RIp_SEM data. Off-diagonal correlations are weak, and it is obvious that mixed MFC items do reproduce the original single-stimulus estimates much better than only positively keyed items.

**Table 6.** Mixed Items: Correlations Between Original Data (N = 601), the Random Ipsative Data Weighted 1 and the Random Ipsative Data Estimated Using SEM.

Variable	Neuroticism	Extraversion	Openness	Agreeableness
Extraversion	−.01			
Openness	−.02	.11		
Agreeableness	.10	.08	−.18	
Conscientiousness	−.02	.12	.10	.00
Extraversion_1	−.15			
Openness_1	−.08	−.19		
Agreeableness_1	−.10	.38	.02	
Conscientiousness_1	.03	.01	−.02	.14
Extraversion_SEM	−.41			
Openness_SEM	−.29	.29		
Agreeableness_SEM	.17	.06	−.33	
Conscientiousness_SEM	.06	.21	−.07	.04

*Note.* SEM = structural equation modeling.

**Table 7.** Mixed Items: Correlations Between Original Data and Random Ipsative Data.

Variable	E	A	C	N	O
Extraversion_1	.79	−.15	−.06	.03	.05
Agreeableness_1	−.03	.71	−.12	.26	.02
Conscientiousness_1	−.02	.01	.78	−.08	−.01
Neuroticism_1	.01	.25	−.06	.84	.09
Openness_1	−.02	.11	.04	.05	.82
Extraversion_SEM	.73	−.15	−.14	.15	.03
Agreeableness_SEM	−.28	.62	.18	.03	.22
Conscientiousness_SEM	−.13	.06	.76	−.29	−.04
Neuroticism_SEM	.13	.19	−.17	.85	.04
Openness SEM	.02	.09	.04	.01	.81

*Note.* E = Extraversion; A = Agreeableness; C = Conscientiousness; *N* = Neuroticism; O = Openness; SEM = structural equation modeling.

### Number of Items

The second set of simulations varies the number of items used. Good reproduction is found using 20 sets of items, but here we also test smaller and larger item sets. To estimate the reproduced correlations, we will estimate the difference between the pattern of correlation in the original data, with the pattern in the reproduced datasets using both RIp_1 and RIp_SEM estimations. We will use sets of 10, 20, 40, 80, and 1,000 MFC items. The simulations will also vary whether items are single-keyed or mixed-keyed. Canonical correlation analysis is used to test how well the methods reproduce the original data. While there is a lot of information in the canonical correlation analyses, only the Pillai trace test of all five dimensions (1–5) will be reported. Pillai’s trace estimates whether all dimensions are related, where a relatively higher number suggests that the variables of the two sets of data are more strongly related.

Table 8 provides the results from the simulations. For several models, it was not possible to get results mostly because of convergence problems for the SEM analysis. For the RIp_1 test based on 10 sets of only positively keyed items, the canonical correlation failed because of singularity in the correlation matrix. The Ip_1 estimation generally performed better than the Ip_SEM estimations, that is, the correlations between the five factors were larger and the canonical correlations were stronger. From the table, it is obvious that the correlation between MFC-data and the single-stimulus data benefited from larger data sets. In addition, it is again clear that mixed items provide better reproduction than only positively keyed items. As a last test we also added an estimation using 1,000 Ip_1 item sets (3000 binary variables). It was found that by using such a large sample it is possible to reproduce the correlations of the original data almost perfectly.

**Table 8.** Simulation of Number of Item Sets in the MFC Data Using the NB5I Inventory.

Item sets	Pillai	E	A	C	N	O
Pos RIp_1 10 sets	Singular	.63	.57	.62	.78	.54
Pos RIp_1 20 sets	2.65	.73	.54	.74	.82	.72
Pos RIp_1 40 sets	3.17	.79	.69	.78	.87	.78
Pos RIp_1 80 sets	3.82	.87	.80	.84	.92	.87
Pos RIp_SEM 10 sets	0.06	.55	.41	.47	.73	.43
Pos RIp_SEM 20 sets	2.48	.63	.26	.23	.77	.60
Pos RIp_SEM 40 sets	No conv					
Pos RIp_SEM 80 sets	No conv					
Mix RIp_1 10 sets	2.30	.71	.60	.60	.73	.64
Mix RIp_1 20 sets	3.14	.79	.78	.81	.84	.73
Mix RIp_1 40 sets	3.82	.87	.84	.88	.92	.84
Mix RIp_1 80 sets	4.23	.92	.89	.91	.95	.92
Mix RIp_1 1,000 sets	4.78	.98	.97	.98	.98	.98
Mix RIp_SEM 10 sets	1.98	.72	.41	.54	.71	.61
Mix RIp_SEM 20 sets	2.97	.74	.73	.79	.84	.74
Mix RIp_SEM 40 sets	3.65	.86	.80	.87	.92	.82
Mix RIp_SEM 80 sets	No conv					

*Note.* MFC = multidimensional forced-choice; E = Extraversion; A = Agreeableness; C = Conscientiousness; *N* = Neuroticism; O = Openness; Pos = only positive; RIp = Random Ipsative; _1 = classic coding; SEM = structural equation modeling; Mix = Mixed.

To create the items based on single-stimulus data sets, the mean ratings of the items need to be rather equal, otherwise some combinations will never be selected. The NB5I inventory is well-suited for this since it was deliberately created to have ratings in the normal population that are close to the midpoint of the rating scale. We attempted to create MFC item sets with the IPIP-300 inventory, but since the mean ratings differ substantially between the items, we ended up with many unselected combinations. The SEM estimations did not converge or resulted in a lot of warnings, but it was possible to use the simple weighting by 1 (Ip_1), the classical coding. Table 9 provides the results from a simulation using the IPIP-300. The correlations between the MFC data and the original were weaker compared with the correlations for the NB5I but rather strong when many item sets were used. Using IPIP-300, it is even more obvious that mixed item sets were an advantage. The last row of Table 9 shows the estimation based on 1,000-item sets, and with such a large data set, the correlations were close to 1.0. Table 10 shows the correlations between the scales in both the original and the MFC using 1,000-item sets. All correlations were not reproduced, but the pattern is similar, i.e., there were moderate correlations between some of the scales.

**Table 9.** Simulation of Number of Item Sets in the MFC Data Using the IPIP-300 Inventory.

Item sets	Canonical Pillai	E	A	C	N	O
Pos Ip_1 10 sets	Singular	.40	.51	.44	.71	.50
Pos Ip_1 20 sets	Singular	.29	.36	.57	.66	.56
Pos Ip_1 40 sets	2.54	.54	.57	.57	.69	.51
Pos Ip_1 80 sets	Singular	.55	.64	.57	.82	.49
Mix ip_1 10 sets	1.72	.63	.55	.59	.73	.55
Mix ip_1 20 sets	2.43	.63	.71	.64	.82	.63
Mix ip_1 40 sets	3.11	.76	.78	.81	.90	.79
Mix ip_1 80 sets	3.69	.87	.85	.83	.92	.81
Mix ip_1 1,000 sets	4.66	.97	.93	.94	.98	.94

*Note.* MFC = multidimensional forced-choice; E = Extraversion; A = Agreeableness; C = Conscientiousness; *N* = Neuroticism; O = Openness; Pos = only positive; _1 = classic coding; Mix = Mixed; IPIP = International Personality Item Pool.

**Table 10.** Correlation Between IPIP-300 Scales Using Original and Mixed MFC Data Based on 1,000-Item Sets.

Variable	Extraversion	Ageeableness	Conscientousnesst	Neuroticism	Openess
Extraversion	.97	.08	.26	.39	.36
Agreeableness	.25	.93	.27	.12	.31
Conscientiousness	.38	.45	.94	.41	.14
Neuroticism	.44	.16	.42	.98	.01
Openness	.45	.44	.31	.14	.94

*Note.* The lower quadrant holds the original correlations, the upper quadrant holds the MFC correlation, and the diagonal the correlation between IPIP-300 and MFC. MFC = multidimensional forced-choice; IPIP = International Personality Item Pool.

### Number of Scales

How many scales can be reproduced using MFC item sets? To test this, we used only the Ip_1 data and attempted to reproduce all 20 facets of the NB5I and the IPIP-300. To make them completely balanced, many sets had to be created (a model that would not be feasible to estimate with SEM), in total there were 1,140 combinations. The correlations between the scales of the original data and the MFC item scales of the NB5I were very strong for all facets. For only positively keyed items the mean correlation was .90 (*SD* = .029) and the mean correlation between facets of the same factors was moderately strong, .30, .30, .38, .56, and .30 for extraversion, agreeableness, conscientiousness, neuroticism, and openness, respectively. The other correlations were mostly slightly negative with a mean of −.13 (*SD* = .11).

Using mixed item sets, the mean correlation was .97 (*SD* = .006), which shows that mixed items is more effective also when the number of variables is larger. The mean correlation between facets of the same factors was higher, .40, .43, .47, .64, and .42, for extraversion, agreeableness, conscientiousness, neuroticism, and openness, respectively. The other correlations were close to zero, *M* = .01 (*SD* = .10). The numbers above can be compared with correlations in the original data, the mean correlation between facets of the same factors were .41, .46, .50, .67, and .43, off correlations were .01 (*SD* = .10).

Using 20 scales from the IPIP-300, the same as are included in the NB5I, we estimated the same model as above. With only positively keyed items, weaker mean correlations for the facets were found, *M* = .80 (*SD* = .06). Also, the mean correlations between facets of the same factors were weaker than for the NB5I, .10, .26, .19, .47, and .12, for extraversion, agreeableness, conscientiousness, neuroticism, and openness, respectively. With mixed items, the mean correlation increased to *M* = .95 (*SD* = .02), and the correlation between facets of the same factors increased to .32, .49, .43, .63, and .30, for extraversion, agreeableness, conscientiousness, neuroticism and openness, respectively. All other correlations had a mean of .16 (*SD* = .18). The values above can be compared with correlations in the original data, correlations between facets of the same factors were .35, .53, .52, .67, and .35, off correlations were .21 (*SD* = .18). It is obvious from these analyses that both the simulated MFC item sets with only positively keyed items and the ones with mixed items have the same information as the original single measure items. When only positively keyed items are used the typical problem with forced-choice items is apparent. Even if it is possible to achieve a high correlation between scales (e.g., between two scales from the same factor), other correlations are affected, that is, they are lower or negative. When mixed item sets are used, the reproduction of the original data is close to perfect.

In the simulation, we used artificial reversed items to make comparisons between the positive and mixed MFC items fair. We also tested using the real positive and reversed items from the NB5I. This inventory is almost balanced with regard to positive and reversed items and as previously mentioned the items are comparable in desirability before reversing (and after). The selection process based on these items resulted in a selection ratio with a mean of .47 (*SD* = .07, range = .31–.63), suggesting small differences in how often an item was selected, even when some were reversed. The reproduction of the original scales was very similar to the artificially reversed versions. For the 80-itemset version, the correlations were .93, .87, .87, .92, and .93, for extraversion, agreeableness, conscientiousness, neuroticism, and openness, respectively.

**Figure 1:**
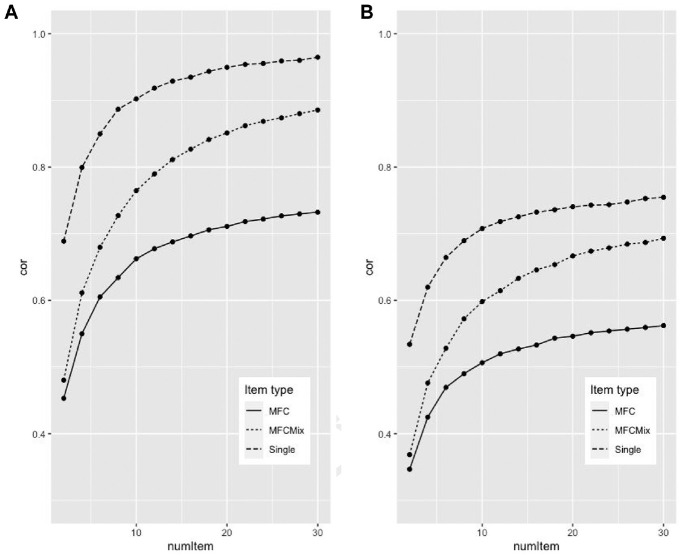
Correlations for the Different Response Formats Across Different Item Set Numbers *Note.* Pane A displays the correlations when items were from the complete inventory. Pane B displays the correlations when items were from a split half inventory, with model and test versions. cor = mean correlation; MFC = Multifactorial Forced Choice; MFCMix = Multifactorial Forced Choice with mixed items; Single = Single Stimulus (Likert-type) format; num Items = number of items in simulation.

Figure 1 shows a direct comparison of how well the different formats can reproduce the correlation in the original data. Two versions were estimated, either we based the items of the complete inventory and correlated the result to this inventory (pane A of Figure 1) or we made two versions of the inventory (split-half) and extracted items from one version and tested it on another (Pane B). The NB5I inventory was used for the simulations, and we only estimated three-factor scales (extraversion, agreeableness, and conscientiousness). The x-axis has the number of items used to estimate each scale. The y-axis has the mean correlation between the traits of the original data and the reproduced data. For two items, the single-item version consisted of six items, and the MFC consisted of two-item sets of three variables. For 30 items, there were 90 items for the Single, and 30 item sets for the MFCs. The MFC line of Figure 1 has the mean correlation for positive/non-mixed item, the MFCmix line has the mixed items, and the Single line are based on a randomly selected items (with replacement) from the total item pool. To estimate the mean correlation, we pooled data from 100 random selection of items for single-item version, and for the MFC items, we pooled 100 random selections. When the number of items was two, the correlations were .42, .44, and .64, for MFC, MFCmix and Single, respectively. The reproduction was much faster for the single-item simulation, than the MFC simulations. However, previous simulation has shown that MFC with mixed items converges close to *r* = 1.0 (see Tables 9 and 10). When using half of the items for creating items and the other one to test, the reproduction was weaker.

Finally, we investigated how well the MFC items reproduce the single-stimulus data, using entirely simulated data. Different sets of data were analyzed, all had 120 single measure items. The amount of correlation between scales was varied as well as the correlation between items of the same scales. In addition, we varied whether the data were based on non-mixed or mixed MFC items. Table 11 provides the mean correlations between scales. Overall, the reproduction was excellent also for the simulated data. Stronger correlations between items from the same variable slightly improved reproduction, and higher correlations between scales also improved the estimations somewhat. Correlations between other scales were mostly underestimated, but not much, and when correlations between scales was close to zero (Lines 1, 6, and 11), this correlation was zero. Table 12 provides the mean correlations for non-mixed item sets. The correlations between scales were rather well reproduced when the correlations between scales was low, but when they increased the reproduction declined. Higher correlations between items provided better reproduction. The correlations between other scales were almost always negative, even when correlations between other scales in the original were very strong.

**Table 11.** Mean Correlation Between Same five Scales in Simulated Mixed MFC Data With Different Patterns of Correlation (240 Binary Items) (K = 10).

Original		Reproduced	
Correlation between items within the scale	Correlation between scales	Same scales	Other scales
.10	.00	.87	.01
.10	.20	.93	.19
.10	.45	.95	.40
.10	.60	.96	.54
.10	.73	.95	.63
.20	.00	.92	.00
.20	.16	.94	.14
.20	.40	.96	.38
.20	.54	.96	.47
.20	.69	.96	.60
.30	.00	.94	.01
.30	.13	.95	.12
.30	.34	.96	.30
.30	.50	.96	.43
.30	.66	.96	.57

*Note.* MFC = multidimensional forced-choice; K = number of simulations.

**Table 12.** Mean Correlation Between Same five Scales in Simulated Non-Mixed MFC Data With Different Patterns of Correlation (240 Binary Items) (K = 10).

Original		Reproduced	
Correlation between items within the scale	Other scales originals	Same scales	Other scales
.10	.00	.80	−.20
.10	.20	.75	−.19
.10	.45	.20	−.16
.10	.61	.53	−.13
.10	.73	.43	−.11
.20	.01	.83	−.21
.20	.15	.78	−.20
.20	.40	.66	−.16
.20	.54	.58	−.14
.20	.69	.47	−.12
.30	.00	.85	−.21
.30	.13	.80	−.20
.30	.35	.69	−.17
.30	.50	.61	−.15
.30	.65	.50	−.13

*Note.* MFC = multidimensional forced-choice; K = number of simulations.

## Discussion

The present simulation studies show that the MFC format reproduced single-item personality estimates very well. When mixed item sets are used there seems to be almost perfect alignment between single-stimulus measures and MFC measures of personality. As was shown in the introduction, there is much more information about the levels (mean values), and how high or low the ratings are on a variable when mixed items are used. This echoes what Schulte et al. (2021) showed, mixed items can be a backdoor to estimating traits in forced-choice inventories. In a sense, this is very strong support for Brown and Maydeu-Olivares’ (2011) idea that personality scales based on single-stimulus measures can be reproduced with MFC item sets, especially mixed ones.

### Measurement Equivalence

The present research started with real-life single-stimulus ratings and from them, we created MFC item sets and analyzed the respondents’ preferences for traits based on how they rated the single-stimulus items. The chief advantage of this strategy is that we know each respondent’s level of latent traits. When simulating an MFC inventory in this way it is possible to estimate how large the discrepancy is in relation to the original data. Surprisingly, the MFC item sets included almost all the information from the single-stimulus measures, and with the preferences, whether they rated a single-item higher or lower than another item, it was possible to reproduce the latent traits almost perfectly. Another thing to note was that when the number of items increased, especially for the mixed item sets, the estimates from the two methods seem to converge. When the number of items increased to a level that would not be possible to use in actual inventories (≥1,000 item sets) the convergence was almost perfect. To us, this suggests that the two estimation techniques are equivalent in relation to what is measured when mixed items are used and that decisions on what technique to use should be based on other considerations. The comparison using completely simulated data showed that non-mixed sets are influenced by the strength of correlation between reproduced sets, making reproductions worse when correlations increased. The opposite was found for mixed items sets. This raises a lot of questions in relation to MFC inventories, but before we discuss these, one cornerstone of the present research needs to be addressed.

### Rationale of the Approach

Maybe the first objection against the present research is that simulated MFC item sets used here are not the same as MFC item sets used in an inventory completed by real participants, although the preferences are taken from real-life single-item ratings. This is of course true, there could be information in the measures that solely stems from the Forced-Choice paradigm. The Thurstonian Factor Models to Forced-Choice Questionnaires that are proposed as useful by Brown and Maydeu-Olivares (2011) describe comparative tasks as discriminatory processes where each item in a set elicits a utility and the item with the highest utility is the one that gets chosen. It is also said that utility is a latent continuous variable that is normally distributed in the population of respondents. In the present research, the utilities are estimated by the respondents’ ratings on the single-stimulus measures of each scale. Brown and Maydeu-Olivares have suggested that the forced-choice instruments can estimate the same latent traits as are estimated by single-stimulus scales; the traits can be reproduced from MFC data. In the present research, we rely on the respondents’ ratings as estimates of item utilities. The big difference is that the utilities from different scales are not compared when issuing the single-stimulus responses. The comparisons are made after the respondents have responded to all items. In the present model, for all randomly created sets, items from three different personality scales are compared, that is, the respondents’ utilities are compared, and the choices are based directly on the ratings. When analyzing MFC data, using the Thurstonian model, the utilities are estimated based on the rankings created by the comparisons. As Brown and Maydeu-Olivares (2011) showed, the convergence between MFC inventories and single-stimulus inventories is quite strong, especially when mixed MFC items are used. The present simulations suggest that this convergence can be even stronger if larger MFC inventories are used.

One problem in our data is that there are a lot of ties, which was solved by randomization, where one item is selected randomly in preference to the others. In real life, having to choose an item over another when they appear to have the same utility probably causes higher cognitive demand, especially when they are matched on social desirability. Our simulated items are easily defended when there are large differences in utilities for different personality factors; the choice is obvious for a person much higher in extraversion than agreeableness. The situation with ties seems also to be very similar in real-life ratings. In relation to the underlying trait, when utilities are equal or almost equal, there should be little information in the choice, it should be almost random. This may be especially so when the latent variables are the goal of the measurement, and less when other choice estimations are done, for example, when choosing among single behaviors, for example, which product to buy. Of course, if we had ratings with more response levels than five, the tie situation would be much more unusual, but when it appears, the solution is the same as in our simulated model, the choices are made randomly. In addition, it was shown that, for mixed items sets, even when the response levels were increased up to 100 the same relation was found between ratings and the sum of the binary data.

### Mixed and Non-Mixed Item Sets

The largest problem with MFC scales, at least the three-item set version that was analyzed in the present research, is that they seem to either estimate the same factors as single-stimulus scales with some problems related to non-independence between scales (when only positively keyed items are used) or estimate the same factors with independence but a need for a very large number of items to reproduce the information. There are many reports of high correlation between MFC and single-item scales, many times so high that the room for unique variance in the MFC inventories is limited. The present research has shown that the mixed MFC response format can reproduce the latent personality traits of single-stimulus inventories almost perfectly. This ability has been suggested as a validation of the forced-choice technique as a measure of traits but is a double-edged sword. If the two techniques are so similar, then, given the many problems with the MFC format, it seems hard to defend its use. The strongest support for this line of argument in the present research is the convergence of the correlational structure when excessively large item sets were used together with mixed items. It seems that all the information from the single-stimulus inventory really can be reproduced/extracted but also that the MFC format tends to add some randomness to the measurement, and this can only be corrected by increasing the number of items. Of course, when small MFC inventories were used, correlation with the single-stimulus measures was only moderate. This could have been suggested to indicate that the MFC also includes specific variance that may be used to estimate interesting criteria better. However, when the number of items increased, this unique variance almost disappeared so an interpretation in terms of non-reliability is more probable. In fact, our direct comparison between random Likert-type items and MFC item sets showed that Likert-type items were more effective, they reliably replicated the structure using fewer items.

However, one of the largest problems with the MFC item technique is the dependence on estimating many variables. The more scales to measure, the less influence from the ipsativity in data when non-mixed MFC is used. So, it is not possible to use MFC when you are interested in only two scales, you need at least three or four. With more scales, there is a need for more item sets. In addition, the only difference between the non-mixed and mixed item sets from the simulation was the lingering negative correlations for non-mixed sets, so it is hard to escape the interpretation that this is artifact, more than substance.

### Does Social Desirability Present the Same Problems Across Methods?

How is social desirability related to forced-choice inventories? When forced-choice inventories are used the norms are not the main target. Rather, the target is the preference for one behavior compared with another. The groundbreaking work by Brown and Maydeu-Olivares (2011) showed that MFC scales could be used to extract the same information as single-stimulus inventories, but ironically, the present work has shown that some forced-choice item formats in fact include information about norms, even when it was not deliberately included. By asking people to compare items from different scales that are keyed both positively and negatively, that is, mixed item sets, and using the binary coding scheme of Brown and Maydeu-Olivares on the responses, the information is there but not in a straightforward way. Brown and Maydeu-Olivares (2011) used SEM to reproduce the traits, but we have shown that it is not necessary to use SEM, simply summing the binary values will extract the information needed to measure the traits. This worked very well, but the problem is that you need a very large number of items to get close to the single-stimulus scale values.

In the simulations, it was shown that the correlation between personality scales from the single-stimulus inventories was intact when mixed MFC item sets were used. This was not the case for non-mixed item sets. There the between-scale correlations were generally underestimated, and for the NB5I they were in fact negative. This alludes to the long discussion about the correlation between Big Five personality scales and how to interpret them. For those who regard the correlations as stemming from the personality structure of people, for example, the GFP, it is probably reassuring to find the same pattern in forced-choice inventories (Pelt et al., 2020). However, there is a large number of studies suggesting that a fair part of this covariation, which is due to measurement factors that can be attributed to the rater (e.g., the GFP, common variance) is not replicated when self and peer ratings of the same person are compared (e.g., Anusic et al., 2009; Bäckström & Björklund, 2014; Danay & Ziegler, 2011). For that group of researchers, it may come as a surprise that the correlations are reproduced also for MFC inventories (Brown & Maydeu-Olivares, 2011). One of the possible causes of the correlations between personality scales is social desirability. When scales of an inventory include a lot of evaluative items the correlations between scales tend to increase. It has been shown that the IPIP-300 has evaluative items and that the factor scales correlate more. If this correlation is reproduced in MFC inventories, as was the case in Brown and Maydeu-Olivares’s (2011) Study 3, then the response format does not seem to do what it is supposed to, that is, control for social desirability.

Several authors (e.g., Pavlov et al., 2021; Wetzel & Frick, 2020) have suggested that also MFC inventories need to have items that are matched with regard to item desirability. However, for many scales, it is extremely difficult to match positively and negatively keyed items with regard to desirability (more about this below). In the present simulations, we used inventories with neutral (NB5I) and evaluative items (IPIP-300). The results showed that the correlational structure was intact. There was little correlation between the factor scales in the NB5I, while there was a substantial correlation between the factor scales of the IPIP-300. One possibility then is that both single-stimulus measures and MFC measures are affected by item evaluativeness (social desirability) and that it can be controlled for in both formats by changing the items to have similar levels of this factor. Bäckström et al. (2009) showed that evalatively neutralized items in a Big-Five inventory decreases the influence of GFP to almost zero. This indicates that it is possible to control for social desirability with the same technique both in traditional and in MFC inventories. In fact, one relevant result from the present study is that when the simulation process was applied on evaluatively neutralized items, both positive and negative items were selected, with similar probability. On the contrary, this does not solve the problem with more specific desirability stemming from the context, such as in a recruitment situation. There the norms do change and to control for item desirability the items must be adjusted to the context. However, this affects both single-stimulus and MFC inventories. To summarize, if someone really wants to control for social desirability on the item level, the inventories must be adapted, and this applies to both single-stimulus measures and MFC measures. One possibility would be to create items that are neutral in the specific situation and use adaptive test techniques to select items that are optimal. Whether this is possible has not been shown yet.

### Implications for Personality Measurement

The simulations included a comparison between classical coding and estimation by SEM. The results favored classical coding, but Schulte et al. (2021), when comparing the methods more extensively, concluded that SEM provides somewhat better estimations. However, classical coding is much simpler and more quickly estimated. It may therefore be the preferred choice, which is also in line with Schulte et al. (2021). In addition, it is difficult to interpret the standardized loadings in the SEM estimations. They vary considerably, some are weak, for example, .10, some strong, often up to .55 or higher, some are even negative, and it is not apparent what this means. One possibility is that the variability suggests that some items are stronger indicators of a factor/scale, but as the same item set is involved in three different trait estimations, it is not straightforward to interpret the loadings.

There exist several measurement factors, and we must do our best to reduce their influence on the concepts we try to estimate. For example, some people tend to confirm (or dis-confirm) statements and it is necessary to balance scales to control for this acquiescence. Furthermore, reactions to positively phrased items compared with negatively phrased item are also problematic. Very often factor analyses suggest that a scale is influenced by more than one factor, and item phrasing may be the cause. Balanced scales will take care of this problem, but the homogeneity of the scale will decrease, such that more items must be included. Extreme responding is yet another problem, some are fond of the middle alternative, and some of the extreme alternative. When not controlled for this measurement factor can decrease both reliability and validity.

All the above-mentioned measurement factors can create bias, but the most problematic seems to be social desirability. Pavlov et al. (2021) have suggested a selection of items that have the same level of social desirability as the solution. Their study included a small example where the items of a personality inventory were investigated for their rated social desirability value. The results showed that item desirability varied a lot between items. Items that were positively keyed for extraversion, agreeableness, conscientiousness, emotional stability, and openness generally had high values, while the negatively keyed items had low values. What is not mentioned in the study is that the negatively keyed items must be reversed. Reversing negative items is regular practice when single-stimulus measures are created. This is also the case when mixed items are used. The problem is that differences in the social desirability of an item will affect the choices since items are pitted against one another. It is not possible to include a very desirable item together with a very undesirable because then the undesirable will almost never be chosen. A mixed item set includes items that measure extraversion together with non-conscientiousness, and agreeableness, or non-agreeableness, together with introversion and conscientiousness. To estimate the scales, all the negatively keyed items must be reversed (in both MFC inventories and single-stimulus inventories). If desirability is to be controlled at the item level this difference in desirability for positively and negatively keyed items will be a problem when creating the item sets. In this situation the MFC inventory creator must use the non-mixed item set, that is, to create item sets that either include only desirable or only undesirable items. Otherwise, the goal of creating sets comparable in desirability is not possible to achieve. The problem with this is that non-mixed item sets will not be independent and ipsativity will create negative correlation between scales. One possible remedy is to add more scales, as this will decrease their interdependence. However, one of the largest problems with the MFC item technique is the dependence on estimating many variables. So, it is not possible to use MFC when you are interested in only two scales, you need at least three or four, but this will not help with regard to the ipsativity-issue, where even more scales are necessary (maybe up to 30, Bürkner et al., 2019). With more scales, there is a need for more item sets. When estimating the latent traits, this may not be optimal. One additional problem is that variables are not reproduced as well when they are highly correlated, so if you are interested in measuring many facets of a concept, for example, neuroticism, non-mixed items is not an optimal choice.

What is the solution to this dilemma? For single-stimulus measures, it has been shown that evaluatively neutral items reduce the influence of social desirability on a personality measure, and in fact, if evaluatively neutral items are used when constructing MFC inventories it should be possible to create good mixed items sets, where both positively and negatively keyed items are used in the same set. The reason for this is that neutral items have the same social desirability value when estimating both extraversion (positively keyed) and introversion (negatively keyed, to be reversed) so that they can be used together in mixed sets together with items that estimate both openness and non-openness behaviors.

### Limitations

One limitation of the present study is that although the format chosen in the present study is the most commonly used, we have not replicated the results on other MFC item formats (e.g., pairs, triplets, quads). As they all rest on the same kind of logic (Schulte et al., 2021), it may be predicted that the results should generalize quite well. Furthermore, to the extent that there is a difference between single-stimulus measures and MFC measures, this difference is likely to be small, making the need for external criteria for comparing the response formats of less value.

The comparisons above concerned simulated item sets, and it may be asked whether the results would be the same for an actual MFC inventory. Arguably, such a comparison is not necessary as Brown and Maydeu-Olivares (2011) already have shown that the formats correlate very strongly. Our results mirror the result in their study very well, mixed item sets worked much better also in their studies, and correlations were impressive. To create an inventory comparative in length to the ones simulated here would not be feasible. Correlations would increase when the number of items increases, and reliability in the MFC format should increase, resulting in stronger validities, but to test for equivalence would be an overwhelming task. In addition, it is not feasible to estimate very large MFC inventories using SEM. There has already been a number of studies comparing criterion validity between methods and it seems fair to say that some support single-item, and some MFC inventories (see Schulte et al., 2021). Simply adding another study comparing criterion validity and convergence between methods would not be decisive, especially as our hypothesis is that they should not differ. Testing this null hypothesis would require an extensive research program.

### Where Should We Go From Here?

The irony with the attempt to use MFC scales to estimate latent traits is that the mixed item sets worked too well. To our knowledge, the close relation between single-stimulus measures and mixed item sets is something that has been neglected. In fact, knowing that there is so much variability in mixed item sets and that this variability is closely related to the single-stimulus rating scale, should be enough to convince everyone that the two methods really are almost the same. The simulations were added to illustrate that this really is the case and, in addition, to provide a feeling for how well the MFC technique reproduces the assumed latent traits of single-stimulus measures. The simulations further show that non-mixed item sets do not reproduce the traits well, as the correlations between scales become negative (or reduced). The current results suggest that personality traits as they are defined within FFM theory can be measured just as validly with single-stimulus items as with MFC items. The two techniques are equivalent as regard to what they measure, but the single-stimulus model is much more effective. As the single-stimulus format is easy to understand and evaluate psychometrically, we predict that it will continue to be popular. Of course, the MFC technique can be used to measure other concepts, for example, it would be more well-suited to measure personality types or can be used when the preference for personality traits really is the goal of the estimate. In that case, the non-mixed item sets should be preferred, since they tend to keep the preference factor intact.

## Notes

1. For five scales you have the following combinations of triplets: 1 2 3, 1 2 4, 1 2 5, 1 3 4, 1 3 5, 1 4 5, 2 3 4, 2 3 5, 2 4 5, 3 4 5. In this version, the first scale is in Position 1, six times, this will create high mean values for that scale. A balanced version has the following triplets: 1 2 3, 2 1 4, 2 5 1, 1 4 3, 3 1 5, 4 5 1, 4 3 2, 5 3 2, 5 2 4, 3 4 5. All columns have been in every position two times.

2. We conducted many estimations based on the Excel macro created by Brown and Maydeu-Olivares, and always received the same warning messages: “THE RESIDUAL COVARIANCE MATRIX (THETA) IS NOT POSITIVE DEFINITE” and “THE MODEL CONTAINS A NON-ZERO CORRELATION BETWEEN DEPENDENT VARIABLES. SUCH CORRELATIONS ARE IGNORED IN THE COMPUTATION OF THE FACTOR SCORES.” We nevertheless received results, and for all models the fit was very good according to the RMSEA. The CFI did not suggest good fit, but this may be due to the many restrictions in the models.
